# Palestinian Children in the Hemato-Oncology Ward of an Israeli Hospital

**DOI:** 10.4137/cmo.s346

**Published:** 2008-01-21

**Authors:** Miri Nehari, Bella Bielorai, Amos Toren

**Affiliations:** 1 Hemato-Oncology Department, Safra Children Hospital, Sheba Medical Center, Tel-Hashomer, Israel; 2 Hemato-Oncology Department, Safra Children Hospital, Sheba Medical Center and Sackler Medical School, Tel Aviv University, Tel-Aviv, Israel

**Keywords:** pediatric hemato-oncology, staff-patient relationship, splitting, conflict, war medicine

## Abstract

**Purpose:**

An encounter between Palestinian parents of children with cancer and Israeli medical staff is a very special situation where “potential enemies” interact in a caring, trusting and intimate relationship for long periods of time. Our aim was to study the psychological and cultural encounter in order to understand the dynamics involved.

**Method:**

The study is a qualitative one. Data was collected by way of structured in-depth interviews. Participants were physicians and nurses employed in the department, and Palestinian parents accompanying their children who were hospitalized during the research period.

**Results:**

Six main themes emerged from the analysis of the interviews: (1) The decision to come to Israel for treatment. (2) The “meeting points” of the two peoples: the Israeli check points and the Palestinian Authority permits. (3) Encounter with the Israeli hospital. (4) Relationship between medical staff and parents. (5) Language and cultural barriers. (6) Emotions, thoughts and behaviors during high security tension.

**Conclusion:**

The interviews depict a poignant picture of the unique encounter between Israeli Doctors and nurses and Palestinian parents. The psychological mechanism used by parents and doctors is “splitting”-having a dichotomized, simple emotional-perceptual picture of a situation with no conflicts. Nurses use another psychological mechanism in addition which enables them to contain the paradox and the conflict.

## Introduction

The treatment of children with cancer entails accompanying them on a long journey of fear, pain, hope, anger and despair.

The encounter between Palestinian families of children with cancer and Israeli medical staff is, in our view, an exceptional situation of people who are involved in armed struggle and are required to be in a long-term relationships of care involving trust and intimacy. Medical staff that cares for people at war is not a new phenomenon. Most such medical occurrences are characterized by one of the following:

1. Doctors who treat “enemy” patients, mostly in emergency circumstances, where long-term personal relationships are not a factor
2. Doctors who treat people in a war that does not involve their nationality. For example, European physicians who work with war victims in Asia or Africa, whereby interpersonal relationships in such circumstances do not necessarily involve an “enemy”.

At the Hemato-Oncology department at Safra children’s hospital, Sheba Medical Centre, Palestinian children, predominantly from the Gaza strip, have been treated during the armed struggle “Intifada” years.

For the Palestinian parents who bring their children to an Israeli hospital the psychological implications are complex. They have to entrust the life of their child in the hands of an Israeli doctor who may also serve with the IDF reserve forces. Concerning the Israeli medical staff, the circumstances are just as complex, particularly when a member of their own family may have been killed or injured during a terrorist attack.

The aim of this paper is to investigate and shed some light on the intra-psychic processes and mechanisms used, which make it possible for medical staff and patients who are “potential enemies” to work together under such paradoxical circumstances.

## Method

### Participants

1. **Israeli Medical staff**, physicians and nurses, working at the Hemato-oncology department. All staff members who were approached agreed to be interviewed. The nurses interviewed were those who had worked on the ward for at least 2 years and were on day shifts during the research period. 17 medical staff participated: 7 physicians and 10 nurses.
2. **Palestinian Parents accompanying** a sick child who was hospitalized in the department during the two-month research period. All parents approached agreed to participate. 12 family members were interviewed: 8 mothers, 3 fathers and 1 grandfather.

### Instrument

The study was designed as a qualitative one, and phenomenological, structured in-depth interviews were done to collect the data. The structured in-depth interview was designed to enable participants to give a full and detailed picture of their narratives and the processes involved (Patton, 1990). Two different forms of the interview guides were developed: one for Israeli medical staff and one for the Palestinian adult members accompanying the sick child. The interviewees were asked in an interactive informal atmosphere to share their thoughts, feelings and observations about the topics raised (Moustakas, 1994).

### Procedure

All the interviews were conducted in the ward. Medical staff interviews were conducted in Hebrew and lasted 30 to 40 minutes. Parents were interviewed in Arabic by an Israeli Arab social worker, not a member of the department staff. The interviews lasted between one and a half to two and a half hours each.

At the beginning of each interview, the aim of the study and its methods were explained to the interviewees. It was stressed that they could refuse to participate, or choose to stop the interview at any time. The interviews were conducted in a supporting, empathetic and respectful atmosphere (Lincoln, 1995).

All interviews were recorded. The ones conducted in Arabic were translated into Hebrew. The interviews were content analyzed by methods influenced by Straus and Corbin’s (Strauss and Korbine, 1990) work.

The aim of the analysis was to find common content themes and to delineate meanings and common representations of life situations (Maxwelle, 1996; Lincoln and Guba, 1985).

## Results

The interviews of the parents and those of the medical staff were analyzed separately and will be presented separately.

### Part 1: Results of the Parents’ Interviews

The analysis generated six main themes, some of which have sub-themes.

#### The decision to come to Israel for treatment

Although we expected this decision to be a psychologically difficult one, this was not the case.

-“*There were no hesitations* (about treatment in Israel) *the sick girl’s life left no room for it. The only fear was for the girl’s life*”.

#### The border checkpoints and Palestinian Authority permits

This point on the chronological narrative is no doubt the major problem, emotionally and behaviorally. It consists of getting the Palestinian Authority permits to come to Israel for treatment, and the actual border crossing. The parents did not hesitate to express feelings of anger, distress, powerlessness, fear and humiliation. They drew, without restraint, a very unpleasant picture of the difficulties in acquiring Palestinian Authority permits to come for treatment, and even more so of the behavior of Israeli soldiers at the border.

-“*At times of military tension, it is torture to get permit papers* (from the Palestinian authorities) *and torture to cross the checkpoint*.”

#### At the oncology ward

##### The staff’s behavior with patients and parents

In all the interviews, without exception, the parents said that the behavior of the physicians and the nurses towards them was exceptionally good. They said the staff went out of their way to make them feel accepted and to ease their fears of the illness, the treatments and the new, unknown hospital.

-“*What made it easier at the beginning was the incredible attitude of the nurses and the staff. They enveloped me with care and warmth*.”

All the parents described the staff as warm, caring, professional and inspiring trust and hope, treating Palestinian children as they treat Israelis.

-“*The professionalism, the caring, the respect are constant, always there. I don’t see that their attitude changes even when I look at the sad reality in the world outside and around us*”

-“*It crossed my mind that they* (the staff ) *would hate us. Now I know it is not true*”.

##### Medical conditions

All the parents mentioned the excellent medical conditions, the wonderful facilities and the promptness, efficiency and professionalism with which they were received.

-“*Immediately upon our arrival, we were sent for very professional meticulous tests. There was an atmosphere of caring, trust and respect, and everything is so clean and efficient and organized*.”

#### Times of high military tension

We expected the parents to be reluctant to talk about this subject, but this was not the case.

##### Apprehension at the time of a “Pigua”-terrorist attack

Some of the parents expressed fear that the staff would not want to treat them when tension rises or a terrorist attack occurs, realizing later that this does not happen.

-“*Once when there was an attack, I was afraid that I will be sent away without receiving treatment. But this did not happen. We were surprised and relieved that all went quietly*.”

##### Separating the hospital from the outside

The parents described a clear separation between being insight the hospital, where it is safe, and the outside, where it is not.

-“*I can state the hospital is a different world. There is no interest in the outside and what is happening there. If anything happens it does not affect the attitude of the staff towards us*.”

The parents separated between what was happening in the country politically, which they felt was not relevant, and what was happening in the treatment of the child, which was the only relevant issue.

“*I distanced myself from anything political; I detached myself from anything that was not relevant to the treatment of my son’s illness*.”

#### Cultural differences and being away from home

All the parents mentioned the language barrier as the main issue that created a distance and feelings of being a stranger, for the parents as well as for the children. The language issue made it difficult to relate to the staff as well as to the Jewish families.

Cultural differences were mentioned, for example, tastes and smells of Israeli foods which they dislike. Their difficulties at feeling the sanctity of the Muslim Friday in the hospital. The parents expressed strong feelings of loneliness and the difficulties stemming from being away from home and the family. But all of them agreed that those difficulties dwarfed in comparison to the fight for the child’s life.

-“*The longing for home for my husband, for my other children is devastating*.”

### Part 2: Results of the Medical Staff Interviews

Following are the main themes that emerged in the analysis of the medical staff interviews.

When analyzing the responses we found that, on some issues, there was a meaningful difference between the responses of the physicians and those of the nurses. We will first present the common responses of the medical team and then add the nurses’ specific ones.

#### Relationship with a Palestinian child

##### Separation between the medical situation and the military one

All the physicians, without exception, and some of the nurses emphatically said that their medical work and the military-political situation are separate matters, and that the one does not, in any way, affect the other.

-“*There is the outside* (the military situation*) and the inside* (the hospital) *and they do not connect*.”

##### Compassion for the Palestinian child and family

The Doctors see the Palestinian child as a patient and are “blind” to his/her nationality. However they were not “blind” to the nationality when relating to the difficult conditions the family had to endure, expressing sadness at their complex plight.

-“*I feel sad for them. It is an impossible situation for them*”

##### Language and cultural barrier

All the physicians and all the nurses spoke of the difficulties created by the language barrier.

-“*I have difficulties in my relationship with the child because of the language. I am also not sure that I read the signs* (of pain) *correctly because of the cultural differences*”

##### The nurses’ complex picture

Most nurses, in addition to their feelings of compassion for the Palestinian child and their separate worlds, inside the hospital and outside it, also described feelings of fear, anger and difficulty when working with the Palestinian children. They, however, stressed strongly that being with a Palestinian child does not in any way affect their work and that they treat the children as professorially as they treat the Israeli ones.

-“*It is not nice to say so, but it is not human not to feel angry or to be afraid of the Palestinians*”

-“*Sometimes when they accept a Palestinian child over an Israeli one* (The policy of the ward is that decisions are made on a medical basis only), *it pisses me off* ”

-“*I try hard to make sure that they are getting the best treatment there is, because I believe that maybe they think that they are being discriminated against*”

#### When a child dies

We asked the staff about their feelings when a Palestinian child dies. All the medical staff’s first reaction was that “*there is no difference between the feelings when a Palestinian or an Israeli child dies.*” Some of them added that due to the language barrier, relationships with Palestinian children were sometimes less intimate.

-“*with one child* (Palestinian) *I was the one to clean the body just after he died and I was working and crying and his grandfather came and wiped my tears*” (said a nurse).

#### The relationship with Palestinian parents

Physicians described their relationships with the parents as very good, sometimes as less difficult than with the Israeli parents. The staff hypothesized that it might be related to cultural norms or to the parents’ fear that if they did not behave respectfully, the treatment would be discontinued.

The nurses’ responses paint a more complex and deeply layered picture. In addition to compassion and caring, they speak of emotional burden and inner conflict.

-“*I have no interest or need to develop a close relationship. There is a certain mechanism of shutting down. I need to do the very best I can, but no more*”.

-“*I don’t have to like Palestinians in order to totally give myself to a child, like any other sick child* ”.

#### When there is high military tension

All the physicians and all the nurses without exception said that on a day of a terrorist attack their behavior when working with Palestinian children did not change and that military tension had no effect on their professionalism.

-“*I make a complete split between my attending to medical issues and a terrorist event, just like the split between work and home, which do not mix*”

-“*I entered the room to treat a child and the television was on an Arabic channel, showing Israeli tanks-a hate program. I had no difficulty treating the child. I was more aware of their uneasiness*” (a nurse said)”.

Some nurses described having negative feelings, which did not affect their professional behavior, but were there nevertheless.

-“*When there is a terrorist event and I am at work, I am stressed always*”.

-“*I was in the ward and there was a terrorist attack and I felt so angry. It was very difficult* ”.

#### The complexity of the situation

A few of the staff interviewed pondered philosophically, somewhat sadly, about the situation.

-“*this is surreal, a theater of the absurd* ”.

-“*Sometimes I think of my fight for his life, the superb medical effort we make and I know that this same life might end up with a bullet. It is an absurd situation*”

-“*we have a nurse on the staff whose sister was killed in a terrorist attack on a bus. I have never seen her act any differently towards a Palestinian child not even when there is a* “*pigua*”.

## Discussion

The social desirability issue is of importance in this study and needs to be addressed. Is it reasonable to expect the Palestinian parent, whose child is in treatment, to respond truthfully during the interview and to express negative thoughts and feelings? Although we assume a social desirability component to exist, the parents seemed not to be too preoccupied with it. They were ready to give very grim descriptions of encounters with Israelis, even when not directly asked, for example at the checkpoints. We therefore think that one can cautiously accept the positive descriptions as valid reflections of their views.

When we set out on this exploratory research, we assumed that the encounter had the potential to be highly intense, psychologically complicated and not easily understandable. We wanted to understand more and shed some light on the psychological mechanisms used in such paradoxical situations.

The main mechanism that seems to emerge from the analysis of the material and used by most participants is “splitting”. Splitting is a defense mechanism against anxiety that is aroused under situations of pressure when one needs to integrate contradicting perceptions related to the self and/or others. Splitting is a mechanism that separates self and/or others into “all good” or “all bad” evaluations, and has been related mostly to pathological conditions. Recently a number of authors have argued that splitting can take place in normal adult populations as well, but little has been written about it (Dean, 2004; Cheng et al. 1998; Leichsenring, 1999). Dean (Dean, 2004) conceptualized splitting “to be a normal cognitive process that acts to structure the world into predictable categories in order to facilitate perceptions of personal control and adaptive action” (p. 31).

There seem to be two splitting processes taking place in the situation of the study. In the first splitting process, the professional characteristics of a person are “split-off” from his nationality. That is, the physicians see the Palestinian child and his parents only as patients, and any attribute of being a “Palestinian” is split off. In the same way, parents see the doctor only as a doctor, “splitting” from him other attributes such as the fact that he is an Israeli, having served in the army as a soldier, or that he may be angry at Palestinian terrorists.

The second splitting process involves a split between “all good” and “all bad” evaluation of self and others. Physicians and parents seem to perceive each other and their own feeling for each other in positive terms only.

The second mechanism that we found is internal paradox. “Internal paradox^”^ (Ghen, 1992; Pizer, 1992) refers to the experience of holding conflicting, discrepant perceptions of reality. It is emotionally a difficult matter to tolerate a paradox. In this concept, one must overcome the urge to opt for one clear and simple truth and accept that social or emotional phenomena do indeed contain elements which are at odds with one another, without resolution. This mechanism seems to be used mostly by nurses. In the interviews, nurses spoke of their very professional behaviors, while at the same time having a wide range of emotions: care, pity, empathy, anger, fear, uneasiness and a wide array of cognitions towards the parents.

We are not sure about the explanation of this finding. Are the nurses more in touch with their inner selves and their feelings, and can they therefore allow themselves to feel more ambivalent and contain the paradox? Is the nurse’s reaction a result of her much closer and intimate interaction with the patients? Or perhaps the physicians do have a stronger need for control and predictability than nurses, and so their use of the splitting mechanism is more pronounced? Other studies (Kash et al. 2000; Ullrich and Fitzgerald, 1990; Pasacerta and Massie, 1990) have shown that nurses and physicians react differently to stress. Kash et al. found that oncology nurses experience more physical symptoms than physicians do “because they … do not use emotional distancing (diminished empathy) as a defense” (p.6). It seems that nurses use fewer defenses, allowing themselves to “be there emotionally” more than physicians do, but pay a heavy psychological price for it.

## Conclusion

This research focused on a unique situation which evokes exteme responses, but nevertheless us to witness mechanisms that we believe are relevant to other medical situations. How does medical staff deal psychologically with emotionally intense paradoxical situations? What are the psychological mechanisms health care providers use to cope with their close, prolonged encounter with death, and their inability to cure a patient. How do they disengage, distance or “split” their work-world from their after-work-world and prevent emotional carry over?

It seems that there are differences between nurses and physicians in the psychological mechanisms as well as the practices used to promote well-being. It seems to us that nurses allow themselves to “get closer to the fire”, but then get burnt. This should be further explored. Practices that are conducive to promoting well being for physicians and nurses should accordingly be developed. Some might need to learn a better utilization of the splitting mechanism, and some might need help in tolerating paradoxes.
